# A Low-Cost Computational Spectrometer Based on a Trained Sparse Base Matrix

**DOI:** 10.3390/s25030953

**Published:** 2025-02-05

**Authors:** Yanbo Gao, Hejia Pan, Yajuan Sheng, Rui Wen, Yuanhao Zheng, Lin Yang

**Affiliations:** 1 Institute of Frontier and Interdisciplinary Science, Shandong University, Qingdao 266237, China; 2 Institute of Space Sciences, Shandong University, Weihai 264209, China

**Keywords:** computational spectrometer, low-cost broadband filters, decoupling optimization, trained sparse base matrix

## Abstract

Computational spectrometers based on coded measurement and computational reconstruction have great application prospects. This paper proposes a computational spectrometer that has a low cost, is easy to implement in hardware, and has high reconstruction accuracy. The proposed computational spectrometer uses low-cost but highly correlated polymethyl methacrylate (PMMA) material as broadband encoding filters, which could affect spectral reconstruction accuracy. To alleviate this issue, we decoupled the sensing matrix, which is the product of the measurement matrix and sparse base matrix, and subsequently optimized the sparse base matrix independently. Enlightened by the neural network method, an over-complete dictionary was trained based on the public spectral dataset, which was used as the required sparse base matrix for reconstruction. Through this method, we achieved good reconstruction results in simulation. In experiments, the spectrometer prototype can achieve a high-resolution spectral measurements, demonstrating the feasibility of a low-cost computational spectrometer based on the trained sparse base matrix.

## 1. Introduction

With the development of compressed sensing theory, many computational spectrometers based on broadband encoding filters have been produced. These spectrometers use broad spectral filters to encode and compress spectral signals. Combined with a compressed sensing reconstruction algorithm, they can acquire more spectral bands with fewer filter channels [1,2,3]. In the existing research, micro-nano structures such as quantum dots [4,5], photonic crystals [6,7], or metasurfaces [8] are often used as broadband encoding filters. These materials are expensive and require high production processes, which limit the large-scale popularization and application of the product. Therefore, this paper proposes a computational spectrometer that uses a PMMA material with good transmittance, low price, and ease of manufacturing as the broadband encoding filters. Compared with computational spectrometers based on micro-nano structures [4,5,6,7,8], the proposed device greatly reduces production costs.

The original spectral signal must be reconstructed by applying computational algorithms after it has been encoded and compressed. According to the compressed sensing theory, if the signal itself or in a certain transform domain is sparse, it can be reconstructed with a high probability below the Nyquist sampling frequency requirement [1,9,10]. This process essentially restores a low-dimensional measurement value to the original high-dimensional signal by solving an underdetermined equation problem. Currently, mainstream reconstruction algorithms include iterative optimization reconstruction algorithms and deep learning-based reconstruction algorithms.

The iterative optimization algorithms are based on sparse prior knowledge. They can iteratively reconstruct the original signal by solving an optimization problem after projecting the original signal into the sparse domain. Such algorithms are widely used in signal reconstruction for computational spectrometers. An interior-point method [11] was used to reconstruct spectral signals in the computational spectrometer that was studied by Kim [12]. Wu [13] achieved good results in hyperspectral reconstruction by using the disciplined convex programming. Chen [14] used the orthogonal matching pursuit (OMP) algorithm in their computational spectrometer. However, when iterative algorithms are used for spectral reconstruction, the solution efficiency heavily depends on the non-correlation between the encoding matrix and the sparse base matrix. To satisfy this requirement as much as possible, the common method is to use encoding filters with low correlation [6,15]. However, the filters with this property are extremely difficult to design and process. The reconstruction algorithms based on deep learning adopt the data-driven approach. These algorithms perform signal reconstruction by learning the structural characteristics of the signal through many training samples, which can achieve higher reconstruction accuracy [16]. Zhang [17] used a deep neural network (DNN) as a decoder for the computational spectrometer and achieved higher-precision spectral reconstruction than the traditional iterative algorithms. WER-Net, which was proposed by Ding [18], has both coding optimization and reconstruction decoding capabilities, which improves the reconstruction accuracy and optimizes the coding efficiency. Feng [19] proposed a computational hyperspectral camera based on the SFP encoder and neural network decoder, which can reduce the incident angle sensitivity. Although a reconstruction algorithm based on deep learning can achieve better reconstruction results, it places high demands on the storage and computational capabilities of the platform, which makes the embedded integration impossible on many hardware devices and in situ measurements difficult [20].

As a representative iterative optimization algorithm, gradient projection for sparse reconstruction (GPSR) [21] has the advantages of occupying less storage space and consuming fewer computing resources. However, it also has a lower reconstruction accuracy than neural networks, especially when the correlation between encoding filters is high. GPSR is based on sparse prior knowledge. According to the theories of Donoho and Candes [9,10], a sparser representation coefficient of the signal in the sparse domain corresponds to a higher reconstruction quality. Therefore, the choice of the sparse domain is very important. Common sparse transformation matrices include discrete cosine transformation matrices (DCT) [22] and wavelet transformation matrices (DWT) [23]. Although these sparse transformation matrices have a relatively simple structure and low computational complexity, the basic shape of their atoms are fixed and the atomic forms are not rich enough, which makes it difficult to adapt to the characteristics of the spectral signal itself. This paper uses the GPSR algorithm to reconstruct the spectral signals. To further improve the reconstruction accuracy, we decouple the encoding filters and sparse base matrix and individually optimize the sparse base matrix. Enlightened by the neural network method, an over-complete dictionary was trained based on the public spectral dataset, which was used as the sparse basis matrix required for the reconstruction solution. The over-complete dictionary obtained through learning has more atoms and richer forms, which can better match the spectral signal and give the signal a sparser representation. This dictionary can reduce the dependence of the GPSR algorithm on the non-correlation of the encoding filters and improve the reconstruction accuracy.

The paper is organized as follows: Section 2 introduces the basic components of the computational spectrometer designed in this paper and its methodology to achieve hyperspectral measurement. In Section 3, the system model is simulated and the simulation results are presented. Section 4 presents the spectrometer hardware prototype and experimental results. Section 5 is the conclusion of the paper.

## 2. Methodology

**Figure 1:**
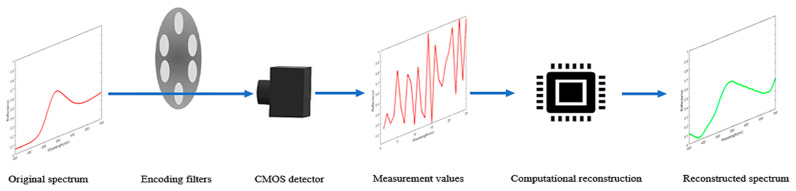
System schematic diagram.

The spectrometer consists of two parts, encoding measurement and computational reconstruction, as shown in Figure 1.

The main components of the encoding measurement part are the broadband filters and CMOS detector. M different broadband filters are installed in a wheel structure. By controlling the filters to switch at a specific rate, the measurement values, $y_{i}$ of the spectral signals under different filters are continuously collected using a CMOS detector,

(1) $$y_{i}=\int_{\lambda_{1}}^{\lambda_{N}}x(\lambda )L(\lambda )R_{i}(\lambda )D(\lambda )d\lambda \;,\;i=1,2\ldots ,M,$$

where x(λ) is the spectral reflectance of the measured target, $L\left(\lambda \right)$ is the spectrum of the lighting source, $D\left(\lambda \right)$ is the spectral response of the detector and $R_{i}\left(\lambda \right)$ is the transmission spectra of different filters.

To perform the spectral reconstruction based on the measured values, Equation (1) must be discretized, and its matrix form is:

(2) y=ϕx,

where $x={\left[x_{1},x_{2},\ldots ,x_{N}\right]}^{T}$ is the original spectral reflectance after discretization; $y={\left[y_{1},y_{2},\ldots ,y_{M}\right]}^{T}$ is the measurement values; ϕ is the measurement matrix of size M×N, Each row of the measurement matrix in this system is obtained from the product of the spectral responses of the light source *L*, i-th filter *R_i_* and detector *D*, i.e.,

(3) $$\phi_{i}=L(\lambda )R_{i}(\lambda )D(\lambda )\;,\;i=1,2\ldots ,M$$

The prerequisite of the compressed sensing theory is that the signal by itself or in a certain transform domain is sparse. Most spectral signals are not sparse in the time domain, so they must be expressed as sparse signals in a certain sparse transformation matrix:

(4) x=ψz,

where ψ is a sparse transformation matrix, and z is the sparse coefficient vector of the original signal x expanded under ψ. Equation (2) can be expressed as:

(5) y=ϕx=ϕψz=Az,

Matrix *A* is the product of measurement matrix ϕ and sparse base matrix ψ, which is called the sensing matrix.

According to the compressed sensing theory, if matrix A satisfies the restricted isometry property (RIP) [24,25], the signal can be reconstructed from a small number of measurement samples. Since the RIP judgment requires many calculations, the commonly used judgment method is the incoherence criterion proposed by Donoho et al. [9]. Here, the row vectors in the measurement matrix cannot be sparsely represented by the column vectors in the sparse base matrix, and vice versa. To try to satisfy this condition, the random measurement matrix is usually constructed by designing many wide spectrum encoding filters and subsequently randomly selecting low-correlation encoding filters from them, which often requires many calculations [26].

### 2.1. Measurement Matrix

According to Equation (3), the measurement matrix is related to the system hardware composition, where the light source and detector remain unchanged. Thus, broadband encoding filters become the key component of the computational spectrometer, which determines the performance of the measurement matrix. Broadband filters usually have high average light transmittance, which helps improve the light utilization and resist the measurement noise interference. Hence, they are usually used as encoding devices in computational spectrometers. In recent years, materials such as quantum dots [4,5], photonic crystals [6,7], and metasurfaces [8] are often used as broadband encoding filters. However, these materials usually require specialized micro-nano design and processing, which are costly and difficult to manufacture, so promoting and applying them on a large-scale is challenging.

PMMA, which is commonly known as organic glass, is a high-molecular polymer material with the advantages of high transparency, low price, easy production and easy processing [27]. Therefore, PMMA materials are selected as the broadband encoding filters of the low-cost computational spectrometer in this paper. These filters are usually made by adding different dyes to the colorless and transparent PMMA material. There is a wide variety of dyes used in colored PMMA. For example, Cromophtal Scarlet R (CGY) is used for red PMMA, copper (II) phthalocyanine is used for blue PMMA, and so on. The production process is simple and low-cost. However, this process makes the transmission spectrum of the PMMA filters insufficiently rich, which is why they have a higher correlation than the micro-nano material filters specially designed for a transmission spectrum. Pearson correlation coefficient correlation coefficients are used to characterize the correlation of filters, which can be calculated by Equation (6).

(6) $$\rho \left(x,y\right)=\frac{\sum_{i=1}^{n}\left(x_{i}-\bar{x}\right)\left(y_{i}-\bar{y}\right)}{\sqrt{\sum_{i=1}^{n}{\left(x_{i}-\bar{x}\right)}^{2}}\sqrt{\sum_{i=1}^{n}{\left(y_{i}-\bar{y}\right)}^{2}}}$$

where *x* and *y* each represent a filter transmittance, $x_{i}$ and $y_{i}$ represent the transmittance corresponding to the *i*-th wavelength of filter curve, $\bar{x}$ and $\bar{y}$ represent the average transmittance of the filter curve. The value of the correlation coefficient ranges from −1 to 1. The smaller the absolute value of the correlation coefficient, the better the non-correlation of the transmittance.

When the transmission spectra of PMMA broadband encoding filters have high correlation, the random selection method cannot ensure that the sensing matrix A satisfies the RIP, and it has limited help in improving the accuracy of the spectral reconstruction. Therefore, in this paper, we decouple the sensing matrix A and subsequently optimize the sparse base matrix using data learning to improve the spectral reconstruction accuracy. This approach avoids several calculations in the design of the measurement matrix and helps reduce the difficulty of the hardware implementation.

### 2.2. Sparse Base Matrix

According to the compressed sensing theory, sparser representation coefficients of the signal in the sparse domain correspond to a higher reconstruction quality. Therefore, the choice of a sparse base matrix is an important factor that affects the accuracy of the spectral signal reconstruction. Commonly used sparse base matrices include the DCT [22], the DWT [23], and the over-complete dictionary [28]. An over-complete dictionary implies that the number of column vectors in the dictionary is greater than the number of row vectors. It is constructed by learning based on the data or the signal itself. Thus, the atoms in the over-complete dictionary are better adapted to the signal itself. An over-complete dictionary can better match the spectral signal and give the signal a sparser representation.

In this paper, we use PMMA filters as broadband encoding devices and the measurement matrix is fixed to reduce the correlation of perception matrix A and improve the reconstruction accuracy. An over-complete dictionary is used as the sparse base matrix. The over-complete dictionary is trained based on the dataset to be sparsely represented. The training process is mainly to minimize the following objective function:

(7) $$\underset{D,C}{\min}{\left\|X-DC\right\|}_{F}^{2}\;s.t.{\;\left\|c_{i}\right\|}_{0}\leq S\;,\;i=1,2,\ldots ,K$$

where *X* is the training dataset; *D* is the over-complete dictionary matrix; *C* is the sparse representation coefficient matrix of the signal, ${\left\|c_{i}\right\|}_{0}$ is the number of non-zero elements in each column of the sparse coefficient matrix; *S* is the sparsity.

### 2.3. Spectral Reconstruction Algorithm

The spectral signal reconstruction process is actually the process of solving Equation (5). When M < N, Equation (5) is an underdetermined equation, and the number of solutions is not unique. Based on the sparse characteristics of the measured signal, the sparsest solution is identified as the best solution. Thus, signal reconstruction is to find the minimum $l_{0}$-norm solution under the constraints of the underdetermined equation. Since the $l_{0}$-norm is not easy to handle, it is necessary to find a new mathematical model to describe the sparsity. Relevant research [29] shows that the minimum $l_{0}$-norm solution can be approximated by the most $l_{1}$-norm solution, and it can be transformed into a convex optimization problem:

(8) $$\underset{z}{\min}\frac{1}{2}{\left\|y-Az\right\|}_{2}^{2}+\tau {\left\|z\right\|}_{1},$$

where the τ is a hyperparameter greater than zero, which is used to control the sparsity of solution.

This paper uses a trained sparse base matrix and traditional GPSR algorithms to solve problems, Equation (8), which can reconstruct the spectral signal and ensure the reconstruction accuracy of the computational spectrometer.

GPSR is a conventional algorithm for solving the convex optimization problem [21]. The algorithm transforms Equation (8) into a quadratic programming problem by representing the unknown quantity *z* as the difference between two non-negative vectors.

(9) z=u−v,u≥0,v≥0,

In this way, Equation (7) can be written as a quadratic programming problem with boundary constraints:

(10) $$\underset{Z}{\min}\;c^{T}Z+\frac{1}{2}Z^{T}BZ\equiv F(Z)\;s.t.\;Z\geq 0,$$

where $Z=\left[\begin{array}{c} u\\ v \end{array}\right],\;b=A^{T}y,\;c=\tau 1_{n}^{T}u+\left[\begin{array}{c} -b\\ b \end{array}\right],\;B=\left[\begin{array}{cc} A^{T}A & -A^{T}A\\ -A^{T}A & A^{T}A \end{array}\right]$.

Using GPSR to solve Equation (8) is to search for the optimal solution in a certain step length along the negative gradient direction until the iteration stops. The iteration formula is:

(11) $$Z_{k+1}=Z_{k}-\alpha \nabla F(Z_{k}),$$

where *α* is step length.

## 3. Simulation Analysis

### 3.1. Simulation Model

To verify the feasibility of the proposed computational spectrometer, we built a simulation model including the original spectral data, sparse base matrix, measurement matrix, and spectral reconstruction algorithm. First, a set of real spectral data with a wavelength range of 400–700 nm and a spectral separation of 2 nm was used as the original spectrum, and a sparse base matrix was used to sparsely represent it. Then, the transmission spectrum data of the PMMA filters were used to construct a measurement matrix, to encode and compress the original spectrum. Finally, the encoded measurement values were input into the reconstruction algorithm for the spectrum reconstruction.

#### 3.1.1. Construction of the Measurement Matrix

**Figure 2:**
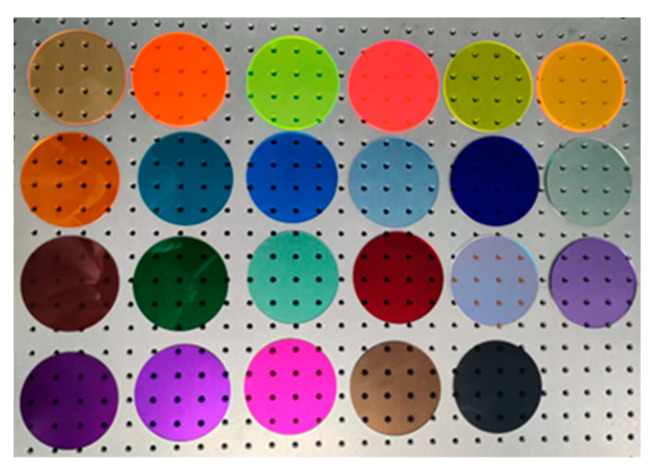
Broadband filters of PMMA.

**Figure 3:**
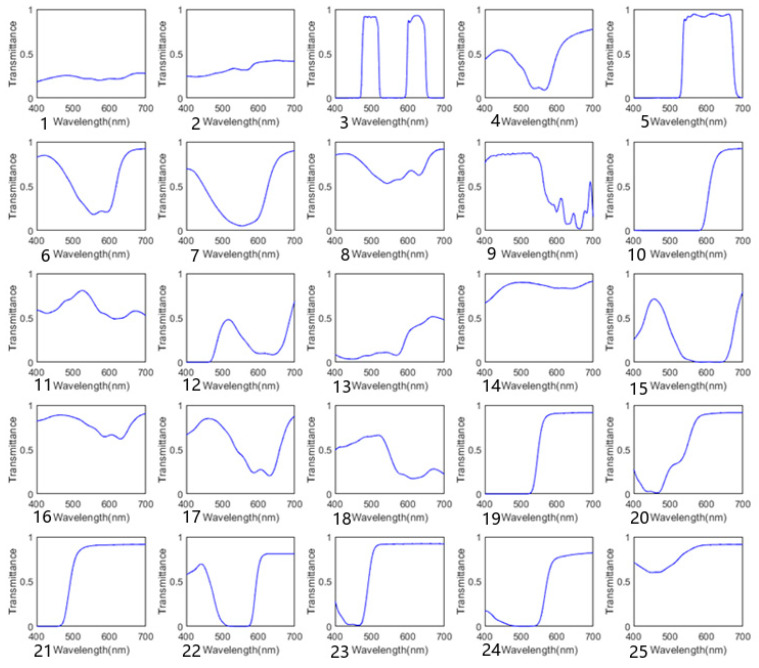
Transmission spectra of the PMMA broadband filters.

We used 25 broadband filters made of PMMA material. Some of the filters are shown in Figure 2. First, the transmission spectra of 25 filters were measured using a Shimadzu UV-3600 spectrophotometer. The transmission spectrum curve in Figure 3 shows that the PMMA filters have a wide spectral response range and high light transmittance. Then, according to Equation (3), the measured transmission spectrum data were used to construct a measurement matrix for the simulation.

**Figure 4:**
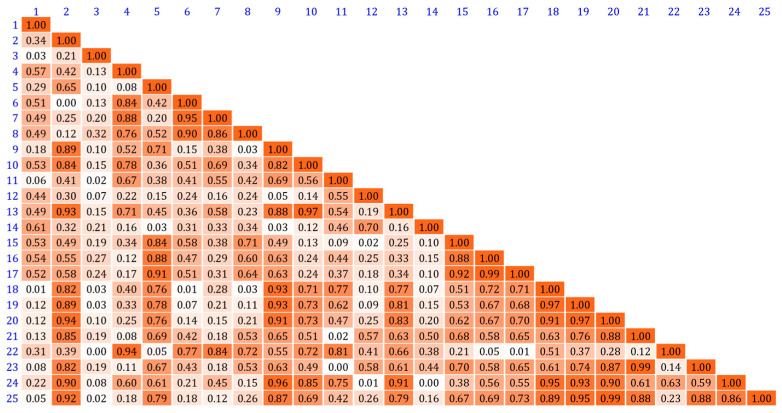
Distribution of correlation coefficients among the transmission spectra of 25 filters.

Based on the discussion in Section 2.1, we quantitatively analyzed the correlation of the transmission spectra of the selected PMMA filters and calculated the correlation coefficients between the transmission spectra of 25 filters. The average correlation coefficient is 0.4631, the highest correlation coefficient is 0.9, and Figure 4 shows the specific distribution. The results show that the PMMA filters used are highly correlated, which introduces challenges to the spectral reconstruction.

When the transmission spectrum of PMMA broadband encoding filters has high correlation, optimization through decoupling is very necessary. In this paper, we decoupled the encoding filters and sparse base matrix and optimized the sparse base matrix using data learning to improve the spectral reconstruction accuracy. Moreover, this approach avoids many calculations in the design of the measurement matrix and helps to reduce the difficulty of the hardware implementation.

#### 3.1.2. Training the Over-Complete Dictionary

Based on the neural network method, an over-complete dictionary was trained based on a public spectral dataset, which was used as the sparse basis matrix needed for reconstruction solution. The spectral data used to train the over-complete dictionary came from CAVE [30] and ICVL [31]. These datasets have relatively rich spectral diversity and cover most of the characteristics of natural spectral data, which helps the resulting over-complete dictionary to have better sparse representation capability, to improve the spectral reconstruction performance.

We used these two datasets to construct a large training dataset, which contained 1.65 million spectral curves in the wavelength range of 400–700 nm and a spectral separation of 2 nm. To reduce the training time and calculation amount, we extracted data from 1.65 million datasets to construct a smaller and representative dataset for dictionary training. First, we calculated the correlation coefficients between each spectral curve and other spectral curves in the 1.65 million spectral data and took the average value. Then, we used this as a basis to divide the large dataset into 1000 distribution intervals. Finally, spectral data were randomly selected at each interval at the same proportion to construct a small dataset that contained 164,540 spectral data. This small dataset contains the characteristics of the entire large dataset to a certain extent. Using it as a training dataset to train an over-complete dictionary can ensure the completeness of the dictionary and help improve training efficiency.

The process of training an over-complete dictionary includes two main stages: sparse decomposition and dictionary update. In the sparse decomposition stage, dictionary D was fixed, the spectral data in the training dataset were sparsely decomposed through the OMP algorithm, and the sparse coefficient matrix C was calculated. In the dictionary update stage, the sparse coefficient matrix C was fixed and each column of the dictionary was sequentially updated using the SVD decomposition algorithm. The training procedure was carried out on a hardware platform equipped with an Intel Core i5-11400 processor.

First, 1000 spectral data were taken from the small dataset of 164,540 to construct an initial dictionary with a size of 151×1000. Then, the process of sparse decomposition and dictionary updating was started to minimize the objective function Equation (7) after the number of training iterations was set.

**Figure 5:**
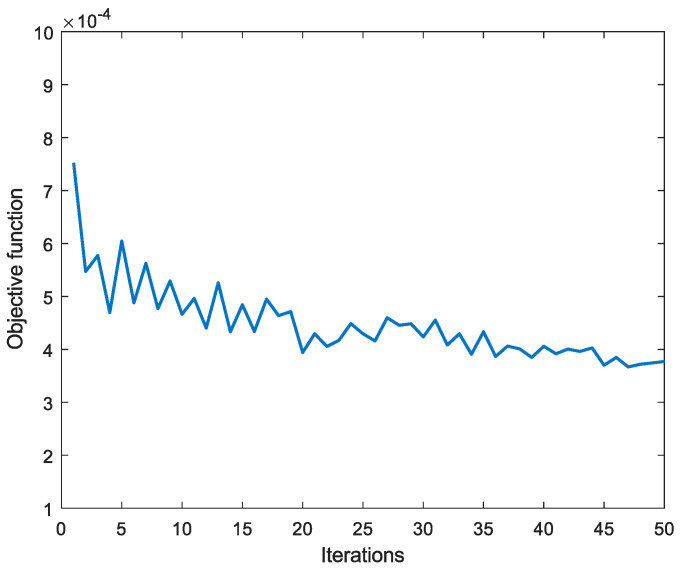
Sparse representation error during the training process.

**Figure 6:**
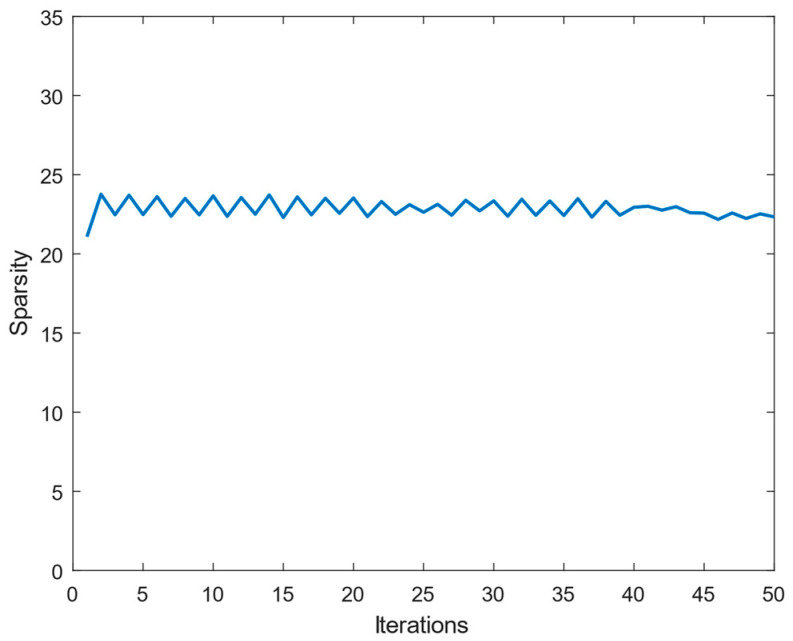
Average sparsity of the sparse representation coefficients.

During the iteration process, Figure 5 shows the change curve of the objective function. The error varied with the number of iterations when the spectral data in the training dataset were sparsely represented by an over-complete dictionary. Figure 6 shows the change in average sparsity with the number of iterations after the spectral data in the training dataset were sparsely represented by the over-complete dictionary. The sparse representation error dropped to the order of below $10^{-4}$ after 50 training iterations, and the sparsity of the spectral signal is higher under the over-complete dictionary. Therefore, using the over-complete dictionary as a sparse base matrix for spectral reconstruction can help achieve better reconstruction results.

#### 3.1.3. Spectral Reconstruction Algorithm

We used the traditional GPSR algorithm for spectrum reconstruction. First, the measurement matrix obtained above and the real spectrum data were used to simulate the encoding measurement process to obtain the measurement values. Then, the measurement values, measurement matrix, and the over-complete dictionary obtained through training were input into the GPSR algorithm. Finally, the algorithm was executed to reconstruct the spectral signal.

**Figure 7:**
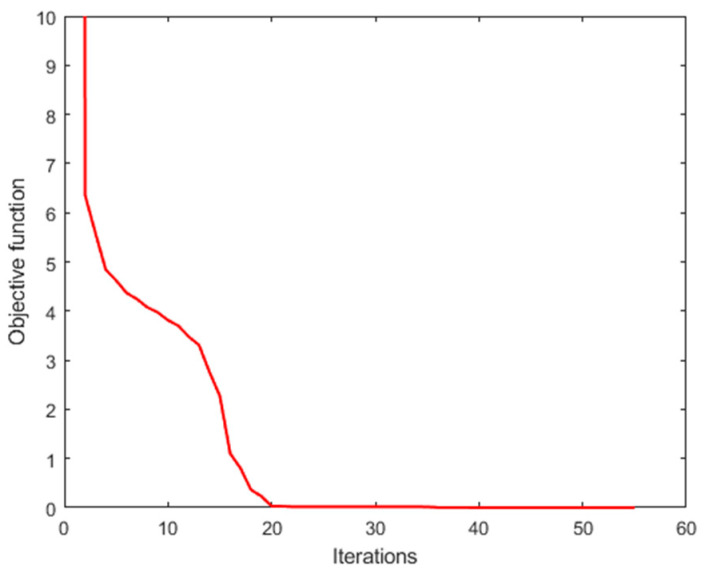
Evolution of the objective function versus iterations.

**Figure 8:**
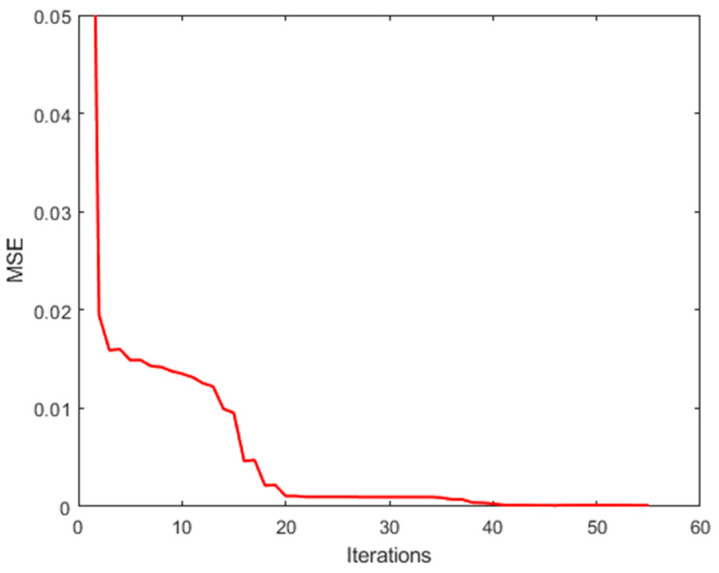
Evolution of the reconstruction MSE versus iteration.

**Figure 9:**
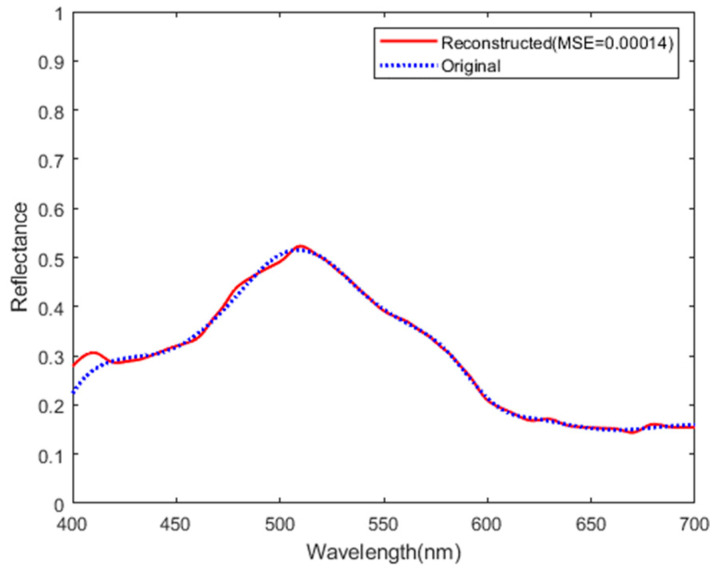
Spectral reconstruction result of the spectrometer simulation model.

The GPSR algorithm minimizes the objective function, Equation (8), through the iterative optimization method. When the relative change in objective function falls below $10^{-3}$, the algorithm is stopped and a sparse solution. In Figure 7, we plot the evolution of the objective function versus iterations. The value of the objective function decreases continuously during iteration and eventually stabilizes. Figure 8 shows that the mean square error (MSE) also decreases and tends to stabilize during iteration. The final spectral reconstruction result is shown in Figure 9, indicating that the proposed spectrometer can achieve good results in simulation.

### 3.2. Results and Discussion

In this paper, we used low-cost but high-correlation PMMA filters as the encoding device of the computational spectrometer, which may have resulted in poorer reconstruction accuracy. To further improve the reconstruction accuracy, we decoupled the encoding filters and sparse base matrix and subsequently optimized the sparse base matrix by training an over-complete dictionary. To verify the effectiveness of this method, we compared the performance of different sparse base matrices in the spectral reconstruction.

**Figure 10:**
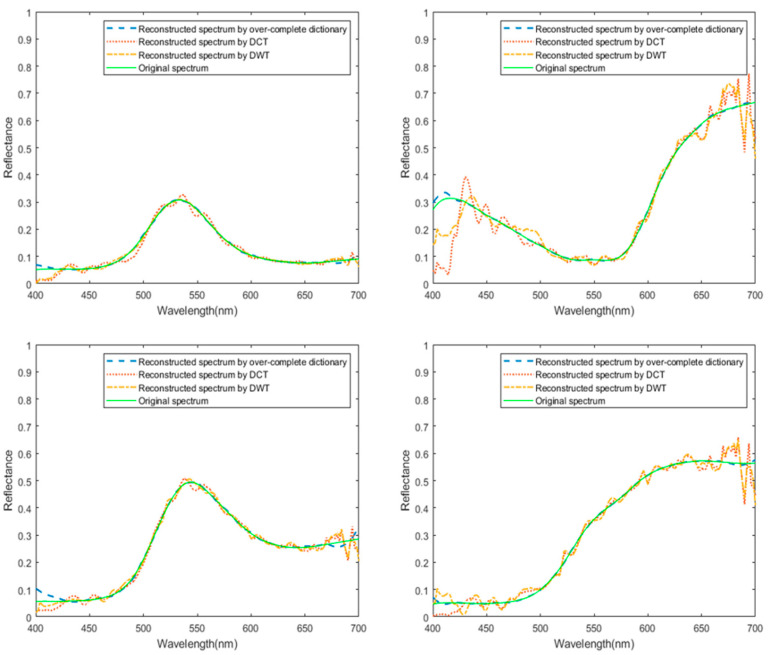
Spectral reconstruction with different sparse base matrices.

Figure 10 shows part of the simulation results. More representatively, we analyze the spectral reconstruction performances quantitatively by calculating mean square error (MSE), peak localization error, peak amplitude error, and full width at half maximum (PWHM). We show the average values of these reconstruction index parameters of all real spectral data used in simulation in Table 1. The simulation results show that when low-cost PMMA materials are used as encoding filters, although the coherence of the transmission spectra between filters is relatively high, an over-complete dictionary trained from the spectral dataset can serve as a sparse base matrix to achieve higher-precision spectral reconstruction with an MSE of $2.65\times 10^{-5}$, which is 132 and 50 times better than those using DCT and DWT, respectively.

**Figure 11:**
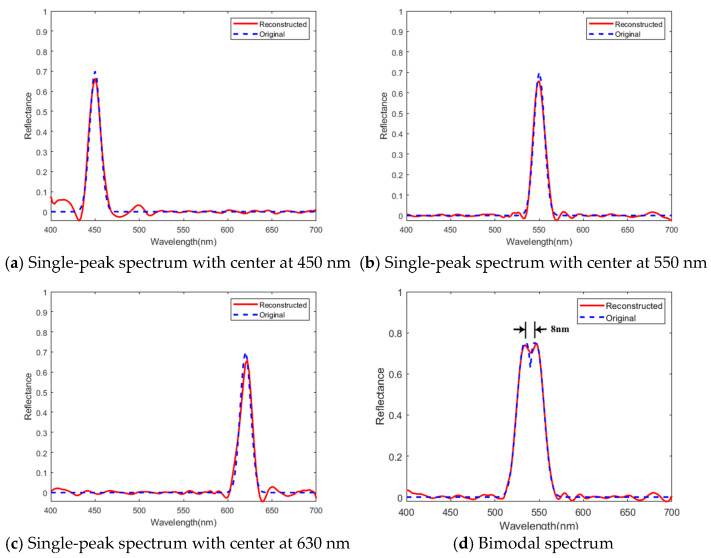
Simulation reconstruction results of narrowband single-peak and bimodal spectrum.

To characterize the spectral resolution of the computational spectrometer prototype in this paper, we used a narrowband single-peak and bimodal spectral curve as the original spectra for simulation verification. As shown in Figure 11, for the single-peak spectra with a full width at half maximum of 8 nm and with the central wavelength at 450 nm, 550 nm, and 630 nm, respectively, the reconstructed spectra have a high degree of agreement with the original spectra. The peak localization error is within 2 nm, and the peak amplitude error is within 5%, indicating that the computational spectrometer has good perception ability for narrowband spectra. The reconstruction result of the bimodal spectrum is shown in Figure 11d. The system can distinguish two peaks with a central wavelength interval of 8 nm, indicating that the spectral resolution of the system is about 8 nm.

## 4. Experimentation

### 4.1. Experimental Method

**Figure 12:**
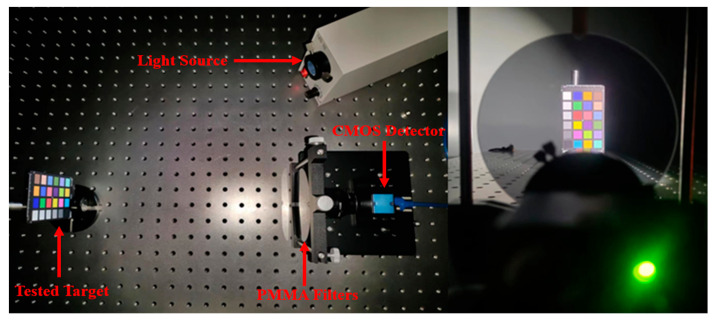
Spectrum collection experimental system.

Figure 12 shows the spectrum collection experimental system. We used the standard color card (model: X-Rite ColorChecker Classic Mini) as the target to be tested. The encoding device is the 25 PMMA broadband filters whose transmission spectra were measured in Section 3.1. We used an industrial CMOS detector (Model: MER-051-120U3M/C) with a 2.8 mm focal length lens to achieve data collection. The light source of the experimental environment is a single LED white light cold light source.

During the experiment, we placed the PMMA filter in front of the detector lens, captured the grayscale image encoded by the filter, and completed 25 spectral encoding measurements by switching the filter. Then, the obtained measurement data were then input into the computer for the spectral reconstruction. According to the simulation results in Section 3.2, we used the GPSR algorithm based on the trained over-complete dictionary to reconstruction, which can enable better reconstruction.

### 4.2. Results and Discussion

**Figure 13:**
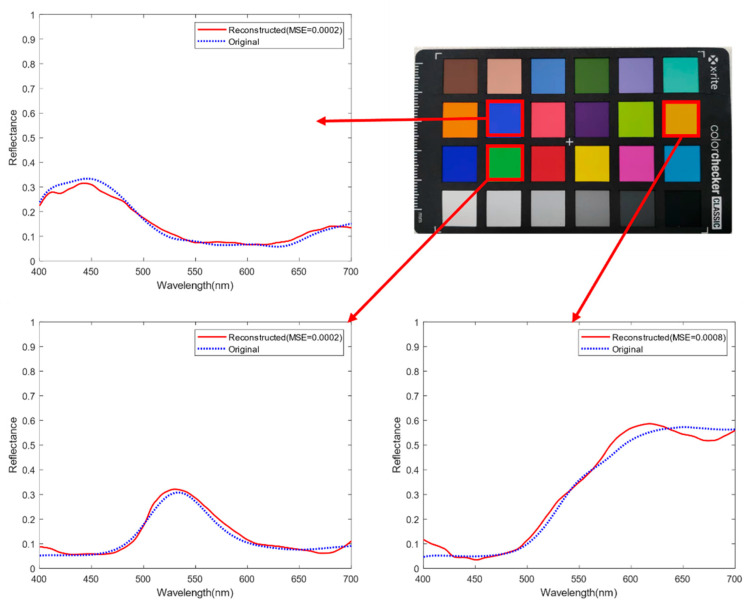
Spectral reconstruction of the color card.

Figure 13 shows the spectral reflectance of different color patches in the 24-color card obtained using the GPSR algorithm based on the trained over-complete dictionary. It can be seen that the spectral trend obtained using this experimental device is relatively close to the real spectral curve and has a good spectral signal characterization effect.

Then, we compared the spectral reconstruction performance of different sparse base matrices in the experiment. Table 2 shows the average values of reconstruction index parameters of different sparse base matrices in the experiment. Although the experimental results are not as good as the simulation results due to the influence of noise and system errors, they still show that using an over-complete dictionary trained from the spectral dataset as a sparse base matrix can achieve higher-precision spectral reconstruction with MSE reaching $2.6\times 10^{-3}$, better than using DWT or DCT.

**Figure 14:**
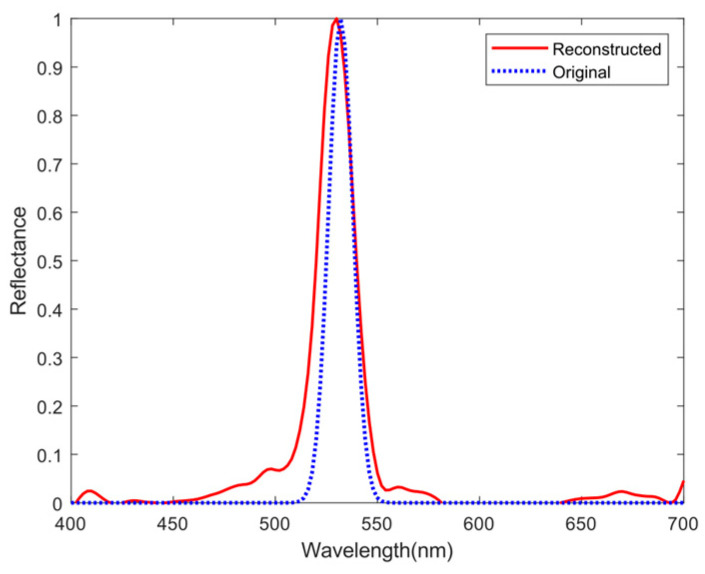
Spectral reconstruction of the monochromatic light source.

**Figure 15:**
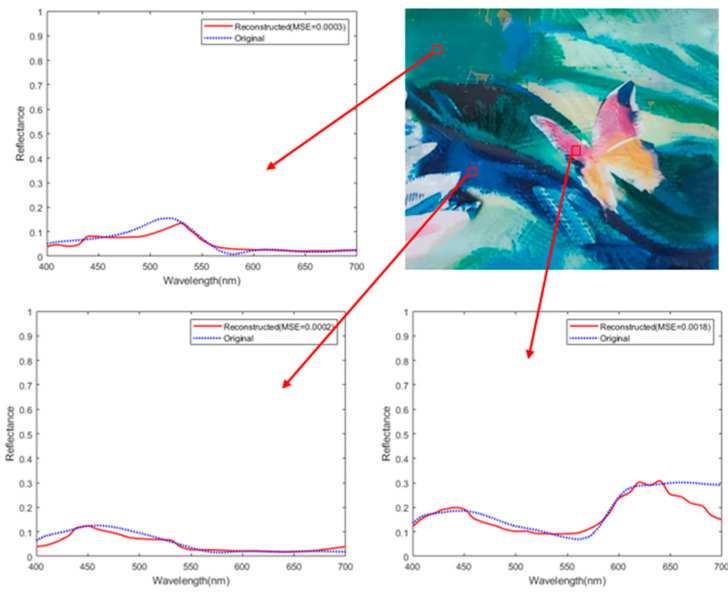
Spectral reconstruction of the packaging paper.

To make the experimental results more representative, we took a monochromatic light source with one narrow peak at 532 nm and the packaging paper of a certain product as the target, respectively, and conducted spectrum collection and reconstruction experiments. The experimental results are shown in Figure 14 and Figure 15. The reconstructed spectra share a consistent variation tendency with the original spectra, and the MSE can reach the 10^−3^ level. For reconstructed spectrum of the monochromatic light source, the peak localization error is 2 nm and the peak amplitude error is within 5%. This indicates that our spectrometer prototype has good measurement capabilities for both narrowband and broadband spectra. Furthermore, we present a comparison table with computational spectrometers based on other structures in Table 3, demonstrating that the computational spectrometer proposed in this study offers a relatively high cost-effectiveness.

## 5. Conclusions

In this study, we proposed a low-cost computational spectrometer based on a trained sparse base matrix and verified its feasibility through experiment. First, a set of low-cost but highly correlated PMMA material filters is used as encoding devices. By switching the filters, we obtained the measurement values of the spectral signals under different encoding filters on the CMOS detector. Compared with computational spectrometers based on micro-nano structures, this method can greatly reduce the production costs. However, the high correlation of the optical filters may lead to poor reconstruction accuracy with conventional iterative algorithms. Enlightened by the neural network method, an over-complete dictionary was trained based on the public spectral dataset, which was used as the sparse basis matrix needed for reconstruction solution. In simulation, the spectral signal was reconstructed through the GPSR algorithm. In the case of a high correlation of the transmission spectra of the PMMA-encoded filters, a higher spectral reconstruction accuracy is obtained by decoupling the sensing matrix and subsequently individually optimizing the sparse base matrix. And the spectral resolution can reach about 8 nm. In experiments, the spectrometer prototype can achieve good spectral measurement results in the spectral range of 400–700 nm, and the average MSE of spectral reconstruction can reach $2.6\times 10^{-3}$. For the reconstruction of narrowband spectra, the peak localization error is within 2 nm and the peak amplitude error is within 5%. The low-cost computational spectrometer proposed in this paper has good measurement capabilities for both narrowband and broadband spectra, which may help promote the application of computational spectrometers with high-correlated optical filters.

## Figures and Tables

**Table 1:** Comparison of the reconstruction performances of different sparse base matrices.

	DCT	DWT	Over-Complete Dictionary
MSE	$3.24\times 10^{-3}$	$1.32\times 10^{-3}$	$2.65\times 10^{-5}$
Peak localization error	9.6 nm	9 nm	2 nm
Peak amplitude error	12.8%	6.04%	2.43%
FWHM error	12.33 nm	9.83 nm	1 nm

**Table 2:** Comparison of the reconstruction performances of different sparse base matrices in experiment.

	DCT	DWT	Over-Complete Dictionary
MSE	$1.11\times 10^{-2}$	$1.35\times 10^{-2}$	$2.6\times 10^{-3}$
Peak localization error	14.2 nm	14.67 nm	4 nm
Peak amplitude error	8.84%	9.42%	4.69%
FWHM error	16.5 nm	12 nm	5.83 nm

**Table 3:** Comparison table with computational spectrometers based on other structures.

	Computational spectrometer proposed in our study	Computational spectrometer based on quasi-random metasurface supercells [14]
Encoding devices	PMMA filters with mature production processes	Quasi-random metasurface supercells specially designed
Spectral resolution	8 nm	6 nm
Cost-effectiveness	Relatively high	Moderate

## Data Availability

Data will be made available on request.
